# The effects of transcranial direct current stimulation on within- and cross-paradigm transfer following multi-session backward recall training

**DOI:** 10.1016/j.bandc.2020.105552

**Published:** 2020-06

**Authors:** Elizabeth M. Byrne, Michael P. Ewbank, Susan E. Gathercole, Joni Holmes

**Affiliations:** a MRC Cognition and Brain Sciences Unit, 15 Chaucer Road, University of Cambridge, Cambridge CB2 7EF, UK; b Department of Psychiatry, Douglas House, 18b Trumpington Road, University of Cambridge, Cambridge CB2 8AH, UK

**Keywords:** Working memory, Cognitive training, Transcranial direct current stimulation, tDCS, Brain stimulation

## Abstract

Transcranial direct current stimulation (tDCS) has been shown to enhance the efficacy and generalisation of working memory (WM) training, but there has been little systematic investigation into how coupling task-specific WM training with stimulation impacts more specifically on transfer to untrained tasks. This randomised controlled trial investigated the boundary conditions to transfer by testing firstly whether the benefits of training on backward digit recall (BDR) extend to untrained backward recall tasks and *n*-back tasks with different materials, and secondly which, if any, form of transfer is enhanced by tDCS. Forty-eight participants were allocated to one of three conditions: BDR training with anodal (10 min, 1 mA) or sham tDCS, or visual search training with sham tDCS, applied over the left dorsolateral prefrontal cortex. Transfer was assessed on within- (backward recall with digits, letters, and spatial locations) and cross-paradigm (*n-*back with digits and letters) transfer tests following three sessions of training and stimulation. On-task training gains were found, with transfer to other backward span but not *n-*back tasks. There was little evidence that tDCS enhanced on-task training or transfer. These findings indicate that training enhances paradigm-specific processes within WM, but that tDCS does not enhance these gains.

## Highlights

• Randomised controlled trial combining backward recall memory training and tDCS.
• Systematic investigation into task features constraining training transfer.
• Measurement of potential benefits of tDCS for training and for transfer across tasks with varying degrees of overlap with training task.
• Training transfer is constrained by paradigm but not task materials.
• tDCS over left DLPFC (1 mA, 10 min) does not enhance training or transfer.

## 1 Introduction

There is widespread interest in the potential for transcranial electrical stimulation (tES) to enhance the effects of cognitive training in a number of domains (Elmasry, Loo, & Martin, 2015), including working memory (WM; Holmes, Byrne, Gathercole, & Ewbank, 2016). While there have been important advances in understanding the underlying mechanisms of tES (Fertonani and Miniussi, 2017, Reed and Cohen Kadosh, 2018) and in testing its effects on a variety of cognitive abilities (Kuo and Nitsche, 2012, Nitsche and Paulus, 2011), the field lacks a detailed understanding of the impact of stimulation on cognitive function. The aim of this study was to provide a systematic investigation of the impact of combining stimulation with WM training on transfer to untrained tasks. By systematically manipulating the overlap in the stimuli and paradigms of the training and transfer tasks, we examined whether the magnitude and distance over which transfer occurs following backward digit span training is increased when it is coupled with stimulation.

Transfer following practice on WM tasks is largely restricted to untrained memory tests that are highly similar to the training activities (Cortese et al., 2015, Gathercole et al., 2019, Melby-Lervåg and Hulme, 2013, Rapport et al., 2013, Redick et al., 2015, Schwaighofer et al., 2015, Shipstead et al., 2012, Simons et al., 2016, Sonuga-Barke et al., 2013, Soveri et al., 2017), although there are some exceptions (Au et al., 2015, Karbach and Verhaeghen, 2014, Klingberg, 2010, Morrison and Chein, 2011, Spencer-Smith and Klingberg, 2015, Titz and Karbach, 2014). To date, there has been little systematic investigation into the processes or features that must overlap between trained and untrained tasks for transfer to occur. The majority of studies in the field rely on post hoc explanations of observed patterns of transfer (e.g. Sprenger et al., 2013, von Bastian and Oberauer, 2013), or include a variety of training activities and/or outcome measures with varying degrees of overlapping task features making it difficult to isolate the task properties that constrain transfer (Anguera et al., 2012, Redick et al., 2013, Sprenger et al., 2013, Thompson et al., 2013, von Bastian et al., 2013). The aim of the current study was to evaluate the magnitude of transfer following training alone (i.e. training without stimulation) to then capture potential enhancements provided by stimulation.

Transcranial direct current stimulation (tDCS) is a non-invasive neuromodulatory tool that delivers weak electrical currents to the scalp to affect processing in the underlying cortex by inducing electric fields. It is polarity-dependent and generates opposing excitatory and inhibitory activity (Nitsche & Paulus, 2000). During anodal (positive) stimulation the excitatory electrode is placed over a location corresponding to an underlying brain region of interest, and a return (cathodal) electrode is placed at a reference location. Anodal tDCS is thought to increase neuronal excitability by altering the resting membrane potential of neurons in the target area (Nitsche & Paulus, 2000). Many studies have explored the cognitive-behavioural benefits of tDCS (for a review, see Nitsche & Paulus, 2011). It has been shown to enhance the efficacy and generalisability of cognitive training in several domains (Elmasry et al., 2015, Hill et al., 2016, Hsu et al., 2015, Mancuso et al., 2016, Shin et al., 2015, Summers et al., 2015). However, there are contrasting reports that tDCS does not induce cognitive change (see Brunoni and Vanderhasselt, 2014, Horvath et al., 2015, Nilsson et al., 2017, Tremblay et al., 2014).

tDCS has been shown to boost WM performance in single sessions (for a review see Hill et al., 2016), but evidence that stimulation enhances WM training is mixed (for reviews see Mancuso et al., 2016, Nilsson et al., 2017). Au et al. (2016) reported enhanced rates of learning (i.e. a steeper rate of improvement) during visuo-spatial *n*-back WM training with active *versus* sham tDCS over left or right dorsolateral prefrontal cortex (DLPFC). Active tDCS also enhanced performance on untrained versions of *n*-back relative to sham stimulation. Similarly, Ruf, Fallgatter, and Plewnia (2017) found that active tDCS to left and right DLPFC enhanced the rate of learning for verbal and spatial versions of *n*-back training, and led to greater improvements on an untrained version of *n*-back relative to sham stimulation. However, other studies have failed to demonstrate enhancements by tDCS. Richmond, Wolk, Chein, and Olson (2014) found that active tDCS over left DLPFC resulted in enhanced on-task training gains on a verbal, but not spatial, complex span task relative to sham stimulation. However, enhanced transfer to untrained WM tasks was only found for the active tDCS group relative to a no-intervention group. Critically, no significant differences were found between the training groups with active and sham tDCS meaning the effects can be attributed to training alone. Martin et al. (2013) reported that tDCS applied over left DLPFC during dual *n*-back WM training did not enhance on-task training gains. In terms of transfer, the training group with active stimulation showed greater gains on an untrained WM task at outcome compared to a tDCS only group (no training), but no significant differences were found between the active and sham stimulation groups (Martin et al., 2013). In another study, Talsma, Lotte, Kroese, and Slagter (2017) found that although tDCS to the left DLPFC boosted performance in the first training session, there were no group differences in overall training gains, or additional benefits to untrained WM transfer tasks, for the active *versus* sham conditions.

Studies investigating the effects of stimulation coupled with WM training are typically limited by one or more shortcomings (Au et al., 2016, Martin et al., 2013, Nilsson et al., 2017, Richmond et al., 2014). First, many fail to use systematic designs to map potential improvements. Due to varying amounts of exposure to each type of training task, it is not always possible to determine which aspects of the training regime mediated observed patterns of transfer. Second, multiple features - stimuli, modality, and recall paradigm - often distinguish the trained and untrained tasks. As a consequence, the possible modulation by training and stimulation of processes associated with particular task features is not tested. Third, most studies combining WM training with stimulation have used *n-*back as the training task (Au et al., 2016, Ke et al., 2019, Lally et al., 2013, Martin et al., 2014, Martin et al., 2013, Ruf et al., 2017, Talsma et al., 2017, Trumbo et al., 2016). Although *n-*back is commonly employed in cognitive training studies, other WM paradigms such as complex span, running span, and backward span are also used. If tDCS is genuinely enhancing the effects of WM training, then benefits should be observed on these other WM training activities. To test this, backward digit recall (BDR) training was used in the current experiment. Backward span is commonly used to measure WM ability in clinical, developmental, cognitive, and educational psychology, but few studies have used it in the context of training. This study is therefore novel in being the first to test the effects of BDR combined with tDCS, and also in being the first to investigate training and transfer effects following practice on BDR alone.

The impact of training and stimulation on transfer for two task features - stimuli and paradigm - were addressed in the current study of WM training with and without stimulation. Participants trained on BDR accompanied by either active (anodal) or sham stimulation and transfer was tested to multiple backward recall and *n*-back tasks. The overlap between training and transfer tasks was varied by WM paradigm (backward recall, *n*-back updating), stimulus domain (verbal, visuo-spatial), and stimulus material (digits, letters). Outcome measures included backward digit (same task), backward letter (same paradigm, same domain, different semantic category), backward spatial (same paradigm, different domain and category), *n-*back digit (novel WM paradigm, same domain and stimuli), and *n-*back letter (different paradigm, domain and stimuli; see Table 1). This design allowed us to investigate the effect of stimulation combined with task-specific WM training on transfer associated with individual overlapping task features. An adaptive visual search (a task with no WM load; Foster et al., 2017, Harrison et al., 2013, Redick et al., 2013) training group was also included. This group trained with sham stimulation to provide an active control for the backward recall sham group, allowing us to conduct a novel systematic investigation into the task features critical to transfer following training on a single WM task.

**Table 1:** Trained and untrained tasks.

Task type	Stimulus domain	Stimulus category
*Training*		
Backward recall	Verbal	Digits

*Transfer*		
Backward recall	Verbal	Digits
Backward recall	Verbal	Letters
Backward recall	Visuo-spatial	Spatial locations
*n-*back	Verbal	Digits
*n-*back	Verbal	Letters

The optimal stimulation montage remains uncertain, with evidence of non-linear dose responses to increases in stimulation intensity and duration (Paulus, Antal, & Nitsche, 2013). Stimulation duration typically ranges from 10 to 30 min, at an intensity of 1 to 2 mA. We applied 1 mA of anodal tDCS for 10 min on the basis that 1 mA leads to greater enhancement of WM than 2 mA (Hoy et al., 2013) and that doubling anodal tDCS from 13 to 26 min converts excitatory tDCS effects into inhibitory effects (Monte-Silva et al., 2013). Stimulation was applied to the DLPFC, a region consistently implicated in WM task performance (D’Esposito et al., 2000, Owen et al., 2005, Smith and Jonides, 1999, Wager and Smith, 2003). The anodal electrode was applied over the left hemisphere to maximise any potential benefits of stimulation with verbal training materials.

The following predictions were tested. The first was that strongest training transfer (without stimulation) would arise when trained and untrained tasks shared a common paradigm (e.g. backward recall with digits to backward recall with letters). The majority of previous studies have failed to demonstrate transfer across different categories of WM task (e.g. Holmes et al., 2019, Li et al., 2008, Minear et al., 2016, Redick et al., 2013, Thompson et al., 2013; although see Anguera et al., 2012, Schwarb et al., 2016, Seidler et al., 2010 for exceptions). These data suggest that transfer is limited by paradigm, and therefore that training benefits are process- or task-specific. A second prediction was that BDR training (without stimulation) would benefit other untrained backward span tasks with verbal stimuli (i.e. backward digits and letters). Backward digit span depends on sets of encoding, maintenance, and retrieval processes applied to verbal short-term memory (Norris, Hall, & Gathercole, 2019). As this system stores phonological rather than semantic representations (e.g., Salamé & Baddeley, 1982), the processes involved in backward recall should be common to letters and digits, so training should benefit same-paradigm tasks containing verbal stimuli equally.

Previous studies investigating within-paradigm transfer to untrained tasks containing memory items from a different domain (e.g. verbal digits to spatial locations) have produced mixed results (Anguera et al., 2012, Blacker et al., 2017, Bürki et al., 2014, Buschkuehl et al., 2014, Holmes et al., 2019, Jaeggi et al., 2010, Küper and Karbach, 2016, Li et al., 2008, Minear et al., 2016, Soveri et al., 2017). Evidence points to a high degree of domain specificity in the mechanisms involved in encoding and maintaining verbal and visuo-spatial short-term memory (Alloway et al., 2006, Baddeley et al., 1988, Della Sala et al., 1999, Pearson et al., 2014), but whether stimulus domain is a barrier to transfer was an open question.

The final training-only prediction was that transfer would not extend to *n*-back tasks following BDR training. Process- and task-specific accounts of transfer predict that it will only occur under conditions where the training and transfer measures engage overlapping processes (Dahlin et al., 2008, Gathercole et al., 2019, Minear et al., 2016, Sprenger et al., 2013, von Bastian and Oberauer, 2013).

In terms of stimulation effects, greater on-task training gains were expected with active compared to sham stimulation, consistent with findings of previous studies combining tDCS with WM training (Au et al., 2016, Ruf et al., 2017). No specific predictions were made regarding the additive effects of tDCS to novel untrained backward recall tasks (with letters or spatial locations), or to *n*-back (with digits or letters) due to inconsistent findings in previous tES studies and the current lack of understanding about how stimulation interacts with learning and transfer (Au et al., 2016, Martin et al., 2013, Nilsson et al., 2017, Richmond et al., 2014, Ruf et al., 2017, Talsma et al., 2017, Trumbo et al., 2016).

The protocol for this study was pre-registered with the Open Science Framework (www.osf.io/r4q3s).

## 2 Method

### 2.1 Participants

Forty-eight right-handed, native English-speaking adults (31 female) aged 18–35 years (*M* = 23.229, *SD* = 3.680) with normal or corrected-to-normal vision completed this study (yielding a power of 0.84 to detect a large effect size, *f*^2^ = 0.35, with linear regression at *p* = .01). Participants were recruited via the MRC Cognition and Brain Sciences Unit, University of Cambridge research participation system or through advertisements within Cambridge University colleges. All participants were stimulation compatible, i.e. they had no history of neurological disease or psychiatric disorder, no history or family history of epilepsy or other seizures, no metallic object(s) in the body, no cardiac pacemaker, no history of head, throat, or brain surgery, and were not taking any drugs that affect the central nervous system (including medication and illicit drugs, excluding alcohol) such as antiepileptic drugs, antidepressants, benzodiazepines, and L-dopa. The study was approved by, and conducted in accordance with, the guidelines of the University of Cambridge Psychology Research Ethics Committee (approval number PRE.2016.016). Written informed consent was obtained prior to testing and participants were paid for taking part.

### 2.2 Materials

#### 2.2.1 Transfer tasks

##### 2.2.1.1 Backward recall

**Fig. 1:**
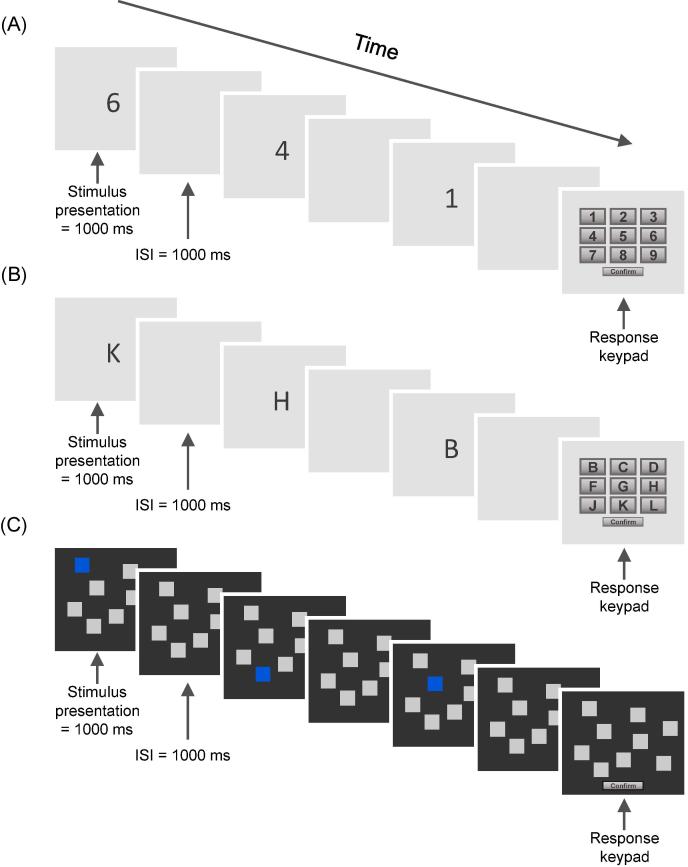
Backward recall tasks (illustrated for a span of 3 items), including: (A) backward digit recall, (B) backward letter recall, and (C) backward spatial recall. ISI = interstimulus interval.

Participants completed three backward recall measures (see Fig. 1), each with a different set of stimuli; (i) digits (1–9), (ii) letters (B C D F G H J K L; i.e. the first nine letters of the alphabet excluding vowels), or (iii) spatial locations (nine boxes at random but fixed locations on the computer screen). Trials were presented in blocks, each consisting of four trials. For each trial the to-be-remembered items or locations were presented visually on screen one at a time for 1000 ms, followed by a blank screen for 1000 ms. Participants were then prompted to recall the sequence in backward order via a touchscreen keypad of digits, letters, or spatial locations, depending on the task administered. Response time was unlimited. All tasks started at a span of three items which increased by one item in each subsequent block if the participant scored three or more correct trials. The task was discontinued if two or more trials were incorrect in a block. Maximum span, as measured by the level the task discontinued on minus one, was recorded.

##### 2.2.1.2 n-back

**Fig. 2:**
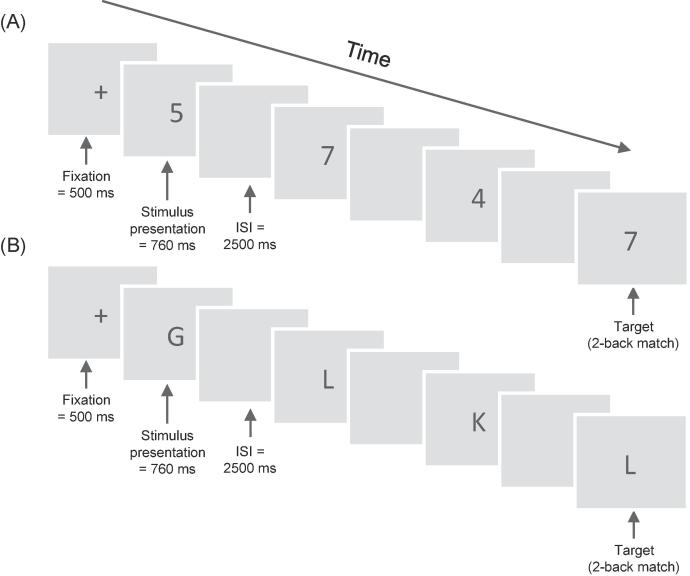
*n*-back tasks (illustrated for a two-back level), including: (A) *n*-back with digits, and (B) *n*-back with letters. ISI = interstimulus interval.

Two *n*-back transfer tasks were administered (see Fig. 2); one with digits (1–9) and one with letters (B C D F G H J K L). Stimuli were presented one at a time in continuous blocks of 20 + *n* items, where *n* corresponded to the number of items back to be matched. Each item was presented for 760 ms, followed by a blank screen for 2500 ms. Participants were required to indicate whether the current item on screen matched the one presented *n* items back in the sequence via a button press. For example, on two-back (*n* = 2) participants had to decide whether the number on screen matched the one presented two items previously in the sequence. In each block there were a total of six possible targets (matches), and 14 + *n* non-targets. Participants were only required to respond to matches and could do so at any time during stimulus presentation or the fixation window for a given trial. An error was scored if participants pressed the button for a non-target (a false alarm), or if participants failed to press the button when a match was present (a miss). Total errors were scored as a combination of false alarms and misses. The first block began at one-back and the difficulty level increased by one in each subsequent block if less than five total errors were made (e.g. increase from one-back to two-back). If five or more total errors were made within a block then the task would end. Maximum *n*-level, as measured by final *n*-level minus one, was scored.

#### 2.2.2 Training tasks

##### 2.2.2.1 Backward digit recall

BDR training involved reverse serial recall of sequences of digits. The stimuli, presentation rate, and response methods were identical to the BDR transfer task (see Fig. 1). This was an adaptive task, meaning the difficulty level was increased or decreased depending on performance. During the first training session the difficulty level was titrated to individual baseline performance (as measured at pre-test) minus one. During the second and third training sessions the task would begin at the highest level reached during the previous training session minus one. The rules for progression up and down the levels within each training tasks were: increase by one storage item if three consecutive correct responses were made, decrease by one item if two consecutive incorrect responses were made, otherwise the sequence length remained the same. Participants completed three training sessions, with 100 trials per training day, yielding 300 trials in total. Using 300 trials is consistent with the number of trials administered for a single training task in Cogmed WM Training (Cogmed. , 2005), one of the most extensively studied programmes that yields large effect sizes for process-specific transfer (Schwaighofer et al., 2015, Sprenger et al., 2013). Average performance, as measured as the average span level reached on correct trials, was scored for each training session.

##### 2.2.2.2 Visual search

**Fig. 3:**
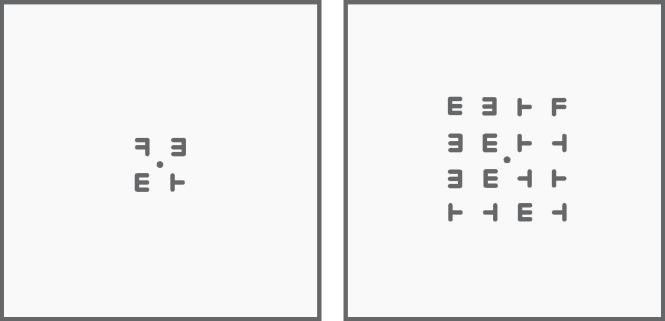
Visual search training task, with illustrations of arrays for level one (left panel) and level five (right panel).

An adaptive visual search task was used as the active control training program (Foster et al., 2017, Harrison et al., 2013, Redick et al., 2013). On each trial participants were presented with a brief array of letters for 500 ms. This array contained a single left or right facing target *F* and multiple distractors made up of left and right facing *E*s and left and right tilted *T*s (see Fig. 3). Participants were then presented with a mask screen for 2500 ms during which time they had to indicate whether the target *F* was facing left or right via button presses. If participants did not respond during this window the trial was scored as incorrect. The difficulty of the task was manipulated by increasing or decreasing the size of the array. Each increase in difficulty alternated between adding another column and then another row to the array. For example; level one was a 2 × 2 array, level two was a 2 × 3 array, level three was a 3 × 3 array, and so on. The rules for progression up and down the levels within the visual search training tasks were: increase difficulty level by one if accuracy in the previous block was equal to or greater than 87.5%, decrease difficulty level by one if accuracy in the previous block was equal to or less than 75%, otherwise the difficulty level remained the same. Each training session began at level one. Participants completed three sessions of training. There were 30 blocks per session, with each block containing 24 trials, yielding 2160 trials over the three training sessions. Average performance, as measured by the average level of difficulty reached across all trials, was scored for each training session.

#### 2.2.3 Stimulation

tDCS was applied to the left DLPFC via two 5 × 5 cm rubber electrodes covered with saline-soaked sponges. An anodal electrode was positioned on the scalp over the area corresponding to region F3 according to standard international 10–20 electroencephalogram (EEG) electrode placement procedure, and a reference cathodal electrode was positioned over the contralateral supraorbital area. Electrodes were secured with a rubber headband and stimulation was delivered using a battery-driven electrical stimulator (DC-STIMULATOR-PLUS; NeuroConn). Participants in the active stimulation group received 10 min of tDCS at 1 mA with 15 s of increasing and decreasing ramps at the beginning and end of stimulation. For those in a sham condition, stimulation faded in for 15 s and then was ramped down over 15 s to mimic the initial sensations associated with actual stimulation and blind participants to their stimulation condition. The display of the simulation machine was identical for active and sham conditions ensuring the participants were blind to the type of stimulation being delivered. The experimenter was blind to stimulation condition for participants in the two BDR training groups but knew participants in the visual search group were receiving sham stimulation. Stimulation was delivered at the onset of training.

### 2.3 Procedure

This was a randomised controlled study. Participants completed the transfer tests in pre- and post-training sessions (average completion time, including short breaks and practice trials = 87.344 min). After completing the pre-training session, participants were assigned to one of three training groups: visual search training with sham stimulation (*n* = 16, 11 female), BDR training with sham stimulation (*n* = 16, 11 female), or BDR training with active stimulation (*n* = 16, 9 female). Stratified randomisation was used to ensure groups were matched for age, sex, and baseline scores on all the pre-training tasks. Randomisation was completed by a scientist who was not involved in data collection. Participants then completed three sessions of adaptive training with active or sham tDCS. Following training participants completed the post-training session. Each testing and training session was completed on a separate day. All were conducted individually with each participant.

### 2.4 Analysis plan

Planned statistical tests were carried out according to our pre-registered analysis plan. To investigate whether participants showed gains on the training tasks, paired-sample *t*-tests were performed separately for each of the three groups. In each case, average performance on training day one was compared to average performance on training day three. Average performance was measured as the average level of difficulty reached on correct trials. A general linear regression was then performed to test whether stimulation (active or sham) predicted differences between the pre- to post-training scores for BDR training. Performance on training day three was entered as the dependent variable, and group (active or sham) and training day one performance were entered as the independent variables.

To test whether training on BDR benefited performance on other backward recall tasks (within-paradigm transfer) and on *n*-back tasks (cross-paradigm transfer); general linear regression analyses were performed separately for each of the five outcome measures. In each case, post-training scores were entered as the dependent variable with pre-training scores and group (BDR sham or visual search sham) entered as the independent variables. Then to investigate whether stimulation enhanced transfer within and across WM paradigms, general linear regressions were conducted separately for each outcome measure with stimulation group as the predictor. In all cases, post-training scores were entered as the dependent variable with pre-training scores and group (BDR active stimulation and BDR sham) entered the as independent variables.

The standard *p* < .05 value was used for determining results of the paired sample *t*-tests used for on-task training gains. A Bonferroni-corrected α level was used in all analyses investigating the transfer of training gains. As there were five outcome measures a *p* < .01 value was used. Null hypothesis significance testing (NHST) was listed as the primary method of analysis. However, recognising the power limitations of the study, Bayesian inference was also used to evaluate the strength of the evidence of training and transfer effects. In contrast to NHST, Bayes factors (BF) quantify the evidence for both the null (absence of training, transfer, and/or stimulation effect) and the alternative hypothesis (presence of training, transfer, and/or stimulation effect), and for this reason are ideal for testing whether the confidence with which null findings can be accepted. BFs are therefore increasingly popular in both cognitive training research and tES studies (e.g. Biel and Friedrich, 2018, De Simoni and von Bastian, 2018, Morgan et al., 2014, Sprenger et al., 2013). Bayesian analyses were exploratory (i.e. they were not stated in the pre-registered report) and were computed in JASP (The JASP Team, 2017) with default prior scales. Inverse BF (BF_10_) are reported to express the odds in favour of the alternative hypothesis (BDR training and/or stimulation has an effect) compared to the null (no effect of BDR training and/or tDCS). By convention (Jeffreys, 1961), values >1 indicate increasing evidence for the alternative hypothesis (H_1_) over the null hypothesis (H_0_), and are interpreted as follows: 1–3 (anecdotal evidence), 3–10 (substantial evidence), 10–30 (strong evidence), 30–100 (very strong evidence), and >100 (decisive evidence). The corresponding values in support of the null hypothesis are the inverse values (smaller than 1): 0.33–1.0 (anecdotal evidence), 0.10–0.33 (substantial evidence), 0.03–0.10 (strong evidence), 0.01–0.03 (very strong evidence), and <0.01 (decisive evidence).

## 3 Results

### 3.1 Training

**Fig. 4:**
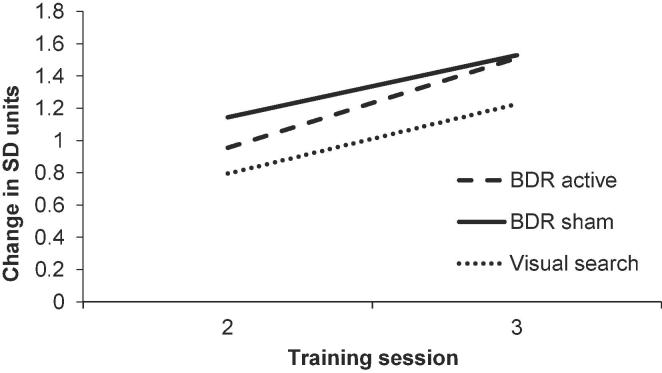
Average difficulty level attained on each session was converted to standard deviation (*SD*) units relative to session 1; i.e. (session 2 average - session 1 average)/session 1 *SD*; (session 3 average – session 1 average)/session 1 *SD*. BDR = backward digit recall training.

As shown in Fig. 4 all training groups improved over the three training sessions. Means and standard deviations of average performance in each training session by group are shown in Table 2. To examine on-task training gains, paired-sample *t*-tests were performed separately for each training group. Average performance on day three of training was significantly greater than on day one of training for all training groups; visual search with sham, *t* (15) = −3.901, *p* = .001, Cohen’s *d* = 0.903; BDR with sham, *t* (15) = −5.166, *p* < .001, Cohen’s *d* = 0.961; and BDR with active stimulation, *t* (15) = −5.486, *p* < .001, Cohen’s *d* = 1.006. Bayesian *t*-tests provided strong evidence for these improvements (visual search sham, BF_10_ = 56.610; BDR sham, BF_10_ > 100; BDR active, BF_10_ > 100).

**Table 2:** Average performance in each training session by group.

	Session 1		Session 2		Session 3	
Group	M	SD	M	SD	M	SD
BDR active	7.764	1.490	9.187	2.293	10.015	2.793
BDR sham	7.418	1.159	8.744	1.996	9.189	2.336
Visual search sham	4.790	0.574	5.246	0.766	5.494	0.941

*Note.* BDR = backward digit recall.

To test whether stimulation enhanced on-task training gains for BDR, a general linear regression was run with performance on day three entered as the dependent variable, and group (stimulation or sham) and training day one performance entered as the independent variables. Group did not significantly predict performance on day three showing that stimulation did not enhance training (*p* = .589). A Bayesian linear regression favoured the null hypothesis that stimulation has no effect on training (BF_10_ = 0.138).

### 3.2 Transfer

**Fig. 5:**
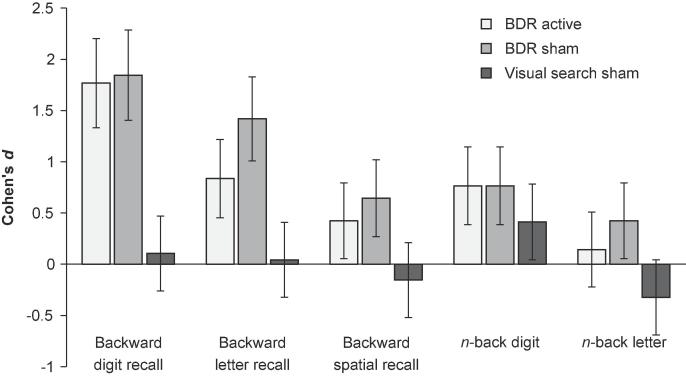
Transfer to untrained tasks. Changes in within-paradigm transfer measures (backward recall tasks) and cross-paradigm transfer measures (*n*-back tasks). BDR active = backward digit recall training with active stimulation, BDR sham = backward digit recall training with sham stimulation.

Changes in transfer task performance in each condition are summarised in Fig. 5 and Table 3. To investigate the effects of training alone on transfer, the BDR sham group was compared to the visual search sham group. A general linear regression analysis was conducted on each of the five outcome measures (see Table 4). In each case, post-training scores were entered as the dependent variable with pre-training scores and group (BDR sham or visual search sham) entered as the independent variables. Greater gains were observed for BDR sham than for visual search sham on the backward digit transfer task (*p* < .001; BF_10_ > 100). Although the NHST group comparisons provided no evidence of transfer, the Bayesian analyses revealed positive evidence for greater gains in the backward letter (*p* = .016; BF_10_ = 3.651) and backward spatial (*p* = .013; BF_10_ = 4.553) transfer tasks for the BDR sham group relative to the visual search group. Group comparisons revealed no evidence of transfer to either *n-*back task (*p*s ≥ 0.06), with the Bayesian regression analyses providing equivocal support for the null and alternative hypotheses (BF_10_s ≤ 1.416).

**Table 3:** Changes in transfer task performance by group.

	Pre-training		Post-training		Pre to post			
	M	SD	M	SD	*t*	*p*	Cohen’s d	Bayesian *t*-test BF_10_
*Visual search sham (n = 16)*								
Backward digit	6.563	1.590	6.750	1.949	−0.527	0.606	0.105	0.289
Backward letter	5.625	1.258	5.688	1.662	−0.144	0.887	0.042	0.258
Backward spatial	5.375	1.204	5.188	1.223	0.588	0.566	−0.154	0.297
*n-*back digit	3.625	1.628	4.625	3.008	−1.711	0.108	0.413	0.843
*n-*back letter	4.063	1.948	3.500	1.506	1.542	0.144	−0.323	0.684

*BDR sham (n = 16)*								
Backward digit	6.313	1.138	10.313	2.845	−7.108	**<0.001**	1.846	>100
Backward letter	5.563	0.964	6.875	0.885	−3.748	**0.002**	1.418	21.677
Backward spatial	5.563	1.209	6.375	1.310	−2.210	0.043	0.644	1.688
*n-*back digit	3.750	1.732	5.688	3.135	−2.565	0.022	0.765	2.925
*n-*back letter	3.688	1.302	4.438	2.128	−1.464	0.164	0.425	0.624

*BDR active (n = 16)*								
Backward digit	6.563	1.459	10.688	2.960	−6.983	**<0.001**	1.768	>100
Backward letter	5.625	1.668	7.063	1.769	−3.216	**0.006**	0.836	8.639
Backward spatial	5.688	1.138	6.375	1.996	−2.033	0.060	0.423	1.304
*n-*back digit	3.750	1.291	5.500	2.966	−2.387	0.031	0.765	2.210
*n-*back letter	4.125	1.408	4.313	1.195	−0.527	0.606	0.144	0.289

*BDR active & sham combined (n = 32)*								
Backward digit	6.438	1.294	10.500	2.862	−10.119	**<0.001**	1.829	>100
Backward letter	5.594	1.341	6.969	1.379	−4.919	**<0.001**	1.011	>100
Backward spatial	5.625	1.157	6.375	1.661	−3.050	**0.005**	0.524	8.469
*n-*back digit	3.750	1.503	5.594	3.004	−3.559	**0.001**	0.776	27.26
*n-*back letter	3.906	1.353	4.375	1.699	−1.507	0.142	0.305	0.525

*Note.* Bold text denote significant effects after family-wise correction for multiple comparison. BDR = backward digit recall.

**Table 4:** Group comparisons of training and stimulation.

	Group comparisons				
	Beta	*t*	*p*	Cohen’s d	Bayesian Regression BF_10_
*Training effects: BDR sham (n = 16) versus visual search sham (n = 16)*					
Backward digit	0.650	5.676	**<0.001**	2.024	>100
Backward letter	0.423	2.551	0.016	0.794	3.651
Backward spatial	0.408	2.632	0.013	0.726	4.553
*n*-back digit	0.158	0.983	0.334	0.347	0.512
*n*-back letter	0.309	1.958	0.060	0.738	1.416

*Training effects: BDR active and sham combined (n = 32) versus visual search sham (n = 16)*					
Backward digit	0.593	6.226	**<0.001**	2.044	>100
Backward letter	0.389	2.984	**0.005**	0.792	9.094
Backward spatial	0.299	2.504	0.016	0.702	3.554
*n*-back digit	0.137	1.024	0.311	0.318	0.485
*n*-back letter	0.270	2.108	0.041	0.638	1.830

*Stimulation effects: BDR active (n = 16) versus BDR sham (n = 16)*					
Backward digit	0.004	0.029	0.977	0.054	0.271
Backward letter	0.610	0.350	0.729	0.078	0.400
Backward spatial	−0.031	−0.201	0.842	−0.088	0.316
*n*-back digit	−0.032	−0.179	0.859	−0.063	0.385
*n*-back letter	−0.098	−0.559	0.580	−0.319	0.413

*Note.* Bold text denote significant effects after family-wise correction for multiple comparison. BDR = backward digit recall.

To investigate the influence of stimulation on transfer within and across WM paradigms, general linear regression analyses were used to compare the BDR training with active stimulation group to the BDR with sham stimulation group (see Table 4). In all cases, post-training scores were entered as the dependent variable with pre-training scores and group (BDR active stimulation and BDR sham) entered as the independent variables. Group did not predict post-training scores for any of the backward recall or *n*-back outcome measures (all *p*s > 0.580). Bayesian regression analyses yielded similar conclusions (all < 1). BF_10_ scores for the backward recall tasks with digits (0.271) and spatial locations (0.316) favoured the null hypothesis that stimulation does not enhance transfer. All remaining BF_10_ scores ranged from 0.385 to 0.413, providing equivocal support for the null and alternative hypotheses (see Table 4).

Transfer to backward letter and backward spatial did not reach significance (difference between BDR sham and visual search sham), but the Bayes factors suggested positive evidence for transfer. Due to low power associated with the relatively small sample size, these could be genuine transfer effects that did not reach statistical significance. To test this possibility the data was collapsed across the BDR sham and stimulation groups to form a larger single BDR (combined) group, and additional exploratory analyses were run. There were no significant differences between the BDR sham and stimulation groups on training and transfer tasks, meaning the groups could be collapsed. By increasing the sample size for this comparison the statistical power was increased for each transfer task (backward digit: from 0.991 to 1.000; backward letter: from 0.225 to 0.403; backward spatial: from 0.178 to 0.297; *n-*back digit: from 0.034 to 0.044; *n-*back letter: from 0.185 to 0.233).

**Fig. 6:**
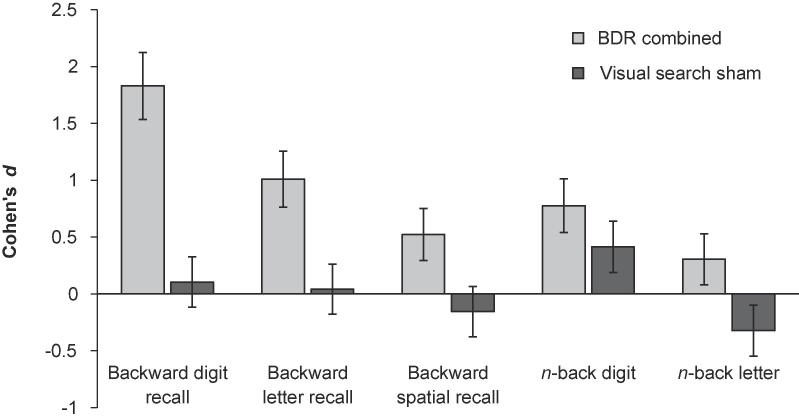
Transfer to untrained tasks. Changes in within-paradigm transfer measures (backward recall tasks) and cross-paradigm transfer measures (*n*-back tasks) for the BDR combined *(n* = 32) and visual search sham (*n* = 16) training groups. BDR = backward digit recall.

Changes on the transfer tasks for the BDR combined and visual search groups are summarised in Fig. 6 and Table 3. General linear regression analyses were conducted for each transfer task (see Table 4). In each case, post-training scores were entered as the dependent variable with pre-training scores and group (BDR combined or visual search sham) entered as the independent variables. Significantly greater gains were observed on the backward digit (*p* < .001; BF_10_ > 100) and letter (*p* = .005; BF_10_ = 9.094) transfer tasks for the BDR combined group relative to the visual search group. Results for the backward spatial task were mixed: the traditional NHST group comparison was not significant (*p* = 0.016), but the Bayesian regression revealed positive evidence for transfer to this task (BF_10_ = 3.554). Group comparisons revealed no transfer to either *n*-back task (all *p*s ≥ 0.06), with the Bayes factors providing equivocal support for the null and alternative hypotheses (BF_10_s = 0.485 and 1.830). Note, although there is stronger evidence for transfer to the *n-*back letters task compared to *n-*back with digits, this is unlikely to reflect a genuine difference in gains between the two *n-*back tasks. The bigger effect size, smaller *p-*value, and larger BF_10_ is likely driven by a drop in performance in the control group rather than a substantial gain for the training group.

## 4 Discussion

This randomised controlled trial is the first to track the potential benefits of coupling tDCS with backward digit span training on transfer to untrained tasks that vary systematically in common and distinct features from the training paradigm. tDCS applied to the left DLPFC did not enhance the magnitude of on-task WM training effects, nor did it modulate additional benefits on transfer tasks in an active stimulation group compared to a sham group. When WM training took place without stimulation, WM paradigm was a boundary condition to transfer but stimulus category was not.

Our data reinforce recent reviews reporting that the cognitive behavioural benefits of tDCS are limited (Brunoni and Vanderhasselt, 2014, Horvath et al., 2015, Mancuso et al., 2016, Tremblay et al., 2014). They are also consistent with outcomes of previous training studies (Martin et al., 2013, Richmond et al., 2014, Talsma et al., 2017) and a recent meta-analyses concluding active tDCS is no more effective than sham tDCS for altering WM training performance (Nilsson et al., 2017). Our study is novel in showing no impact of stimulation for the nearest of near transfer within WM, with untrained tests and training distinguished only by single features (stimuli and WM paradigm). They are also consistent with the pattern of results from our previous study in which tRNS – an alternative method of stimulation – did not enhance WM training or transfer to a wider and less systematically constructed set of transfer tests (Holmes et al., 2016). Together, these studies provide convincing evidence that tES is not an effective tool for enhancing the effects of WM training.

There have been reports of enhanced learning on WM tasks during training with tDCS and of increased transfer effects (Au et al., 2016, Ruf et al., 2017). There are many possible sources of these contrasting findings. Most previous studies reporting positive effects of tDCS on WM training have used *n*-back training tasks (Au et al., 2016, Ruf et al., 2017) rather than backward recall. The impact of tDCS on WM training could be specific to the neural or cognitive mechanisms underpinning *n-*back task performance. Both *n*-back and backward recall assess the ability to simultaneously store and process information, cardinal features of any WM task, but they differ in a number of ways. First, the tasks can be distinguished in terms of retrieval demands: backward recall involves explicit serial recall and retrieval is guided by self-generated cues (Kane, Conway, Miura, & Colflesh, 2007), whereas *n*-back requires recognition and can be completed using familiarity-based responding (Oberauer, 2005). The updating requirements also differ: for *n*-back, the full sequence must be refreshed as items are added and dropped; for backward recall the whole sequence must be maintained and transformed at the point of recall. It is not possible to determine how the effects of tDCS might interact specifically with the different processes involved in *n*-back and backward recall from the current experiment, but it appears that the effects of tDCS on learning during WM training are not global. If they were, then enhancements by tDCS should be observed across both WM paradigms.

Differences in the stimulation parameters might also underpin the inconsistencies. The current study applied 10 min of tDCS at a current intensity of 1 mA, Ruf et al. (2017) used the same intensity as the current study for 20 min, and Au et al. (2016) applied 25 min of tDCS at a current intensity of 2 mA. Although 1 mA of tDCS has been shown to be more effective than 2 mA for enhancing WM performance in a single session (Hoy et al., 2013), the authors stimulated for a longer duration of 20 min and used larger electrodes, meaning current density differed between studies.

The optimal stimulation parameters and conditions for tES are not yet known. It is therefore important to establish both the conditions under which stimulation does and does not lead to enhancements. Here we show that tDCS did not enhance cognitive performance, despite evidence that anodal tDCS at an intensity of 1 mA and for a duration of 10 min is sufficient for producing measurable aftereffects in the brain when stimulating the motor cortex (Lang et al., 2005, Nitsche and Paulus, 2000). Our findings are in line with previous WM training studies showing that 1 mA of anodal tDCS for 10 min (Lally et al., 2013) or 20 min (Talsma et al., 2017) did not enhance cognitive performance. When stimulating cortical areas beyond the motor cortex, higher intensities and longer durations may be needed to enhance cognition. A greater understanding of the neurophysiological mechanisms of stimulation and the impact of different parameters when combined with different training regimes is needed (e.g. current intensity and duration; Woods et al., 2016). Research shows that there are various factors that might contribute to inter-individual variability of responsiveness to tDCS, including age, gender (e.g. due to anatomical differences in bone density), genetics, circadian factors, head and brain morphology, hormone levels, and current brain state (for reviews, see Krause & Cohen Kadosh, 2014; L. M. Li et al., 2015, Ridding and Ziemann, 2010).

The inclusion of an active control training group (visual search) in this experiment allowed us to track transfer effects following training alone and to test for the first time the degree of transfer of backward span training to new stimuli within and across the trained domain. Our results showed graded transfer within backward span. Transfer was substantial for backward span with new verbal material (letters) and marginal for backward span for spatial locations, establishing a high degree of domain-specificity.

Paradigm appears to represent a boundary condition transfer, consistent with many previous studies investigating transfer across different categories of WM task (e.g. Dunning and Holmes, 2014, Gathercole et al., 2019, Holmes et al., 2019, Li et al., 2008, Minear et al., 2016, Sprenger et al., 2013, von Bastian and Oberauer, 2013). While there was evidence favouring transfer to untrained backward recall tasks, there was no evidence for transfer to *n-*back following BDR training, even when it contained the same materials as the training activity (digits). The Bayes factor favouring a small effect of transfer to *n-*back with letters is likely driven by a drop in performance from baseline of the control group, rather than reflecting a genuine training effect (Shipstead et al., 2010, Shipstead et al., 2012). The absence of cross-paradigm transfer, even when the same stimuli are employed, rules out the possibility that training enhances an underlying general WM system (Jaeggi et al., 2008, Klingberg, 2010). If it did, the benefits should extend to any WM task. In combination with the generalization of training benefits to different verbal stimuli when the same paradigm is employed, these results also suggest that training-induced changes are not mediated by the development or enhancement of material-specific mnemonic strategies such as chunking individual digits into familiar number sequences (Minear et al., 2016, von Bastian and Oberauer, 2013).

The transfer of training gains to novel verbal stimuli establishes that the semantic category of stimuli is not a barrier to within-paradigm transfer. These data are consistent with reports of transfer across different materials within the same paradigm (e.g. Holmes et al., 2018; Jaeggi et al., 2010, Küper and Karbach, 2016), suggesting that material-specificity does not constrain transfer when the task structure is held constant between trained and untrained tasks. It is also consistent with a recent proposal that training leads to the development of skill-like cognitive routines for unfamiliar WM tasks. These routines specify the processes required to accomplish particular WM tasks and cannot be readily applied to other WM tasks with different structural properties (Gathercole et al., 2019). A cognitive routine developed for backward recall might, for example, involve successive forward retrievals of diminishing length and peeling off the final item one at a time (Anders and Lillyquist, 1971, Conrad, 1965), whereas *n*-back tasks require the continuous monitoring and updating of a list of storage items to determine whether the current stimulus matches one presented *n*-items back in the sequence. The demands of the two paradigms are distinct and require different sequences of cognitive processes to be co-ordinated for task execution (Gathercole et al., 2019). The present data support this hypothesis and indicate that a routine for backward recall is largely restricted to retrieval processes within the verbal short-term memory system due to the weak evidence for transfer to a backward spatial recall task.

The conclusion of the current study is that when using a rigorous, randomised sham-controlled intervention design, there is no evidence that tDCS enhances the benefits of task-specific WM training. It also establishes that transfer following WM training is tightly tied to the characteristics of the training regimes. Transfer does not extend across global changes in WM paradigm, but it does occur within paradigm for backward recall tasks where it is unconstrained by the category of stimulus materials.

## CRediT authorship contribution statement

**Elizabeth M. Byrne:** Conceptualization, Methodology, Software, Formal analysis, Investigation, Writing - original draft, Writing - review & editing, Visualization, Project administration. **Michael P. Ewbank:** Conceptualization, Methodology, Supervision. **Susan E. Gathercole:** Methodology, Writing - original draft. **Joni Holmes:** Conceptualization, Methodology, Writing - original draft, Writing - review & editing, Supervision.

## Declaration of Competing Interest

The authors declare that the research was conducted in the absence of any commercial or financial relationships that could be construed as a potential conflict of interest.
